# CORESS Feedback: Cases from the Confidential Reporting System for Surgery

**DOI:** 10.1308/rcsann.2024.0074

**Published:** 2024-09-01

**Authors:** H Corbett

**Affiliations:** on behalf of the CORESS Advisory Board

## Abstract

CORESS is an independent charity, supported by AXA Health, the MDU and the Kirby Laing Foundation. We are grateful to those who have provided the material for these reports. The online reporting form is available via the CORESS app and on the website (coress.org.uk), which also includes previous Feedback reports. Published cases are acknowledged by a Certificate of Contribution, which may be included in the contributor’s record of continuing professional development, or which may form part of appraisal or annual review of competence progression portfolio documentation. Contributions from surgeons in training are particularly welcome.

## Late diagnosis of ruptured ectopic pregnancy

### Case 291

As the general surgery registrar, I was called to the emergency department by the on-call locum core trainee covering urology and gynaecology to see a 38-year-old woman with a positive pregnancy test and right-sided lower abdominal pain. I was told that the patient was haemodynamically stable. The core trainee had discussed the patient with the on-call gynaecology consultant, who had requested surgical review to rule out appendicitis before seeing the patient.

When I saw the patient at 2.30am, she was in a side room in the minors’ section of the emergency department with a blood pressure of 50/38mmHg. She had no intravenous access, and was pale and dizzy, having been admitted at 9pm. Since admission, she had experienced lower abdominal pain, distention and a number of syncopal episodes. I transferred her to the resuscitation bay, gained intravenous access, administered fluids, cross-matched four units of blood and inserted a catheter. Her systolic blood pressure recovered transiently to 117mmHg before falling to around 70mmHg, with a tachycardia of 90–150bpm.

I contacted the gynaecology registrar and asked him to see the patient and to discuss her with his consultant. The gynaecology consultant eventually attended and obtained consent from the patient for an emergency laparotomy, subsequently undertaking a right salpingectomy for ruptured ectopic pregnancy. The patient had five litres of blood in her pelvis. Postoperatively, she made an uncomplicated recovery.

#### Reporter’s comments

The covering core trainee had not been trained in cross-specialty cover and failed to recognise a critically unwell patient with clinical signs of a classic gynaecological emergency. Emergency department staff also neglected to flag up grossly abnormal observations to other medical staff. Doctors in training covering specialties other than their own, in an on-call capacity, should be given adequate training in advance.

#### CORESS comments

This is a case in which hierarchy, in addition to poor communication, may have played a role. In a young woman with a positive pregnancy test and abdominal pain, the gynaecology team should have been involved early on and senior review indicated if there was diagnostic doubt. Early ultrasonography may have resolved the diagnostic dilemma and prompted earlier intervention.

## Mismanagement of nasogastric tube

### Case 292

CORESS was alerted to the following case, in the public domain, by the Department of Health and Social Care, and the Association of Surgeons of Great Britain and Ireland. The case was the subject of a coroner’s report with the aim of prevention of future deaths. Details of the case and trust involved have been anonymised in this CORESS report.

A 60-year-old woman was admitted to hospital with a 48-hour history of cramping abdominal pain, vomiting and constipation. The patient had previously required a colectomy for complications of inflammatory bowel disease. She had a distended abdomen with tinkling bowel sounds and examination confirmed the scar of a previous laparotomy. Abdominal x-rays demonstrated distended loops of small bowel with multiple fluid levels and a diagnosis of subacute small bowel obstruction was made.

The patient was placed nil-by-mouth, an intravenous line was set up, and she was catheterised and admitted to the ward for nasogastric tube placement, with an oral request that the tube be aspirated at 2–3-hourly intervals. A request was made for computed tomography and during this, the nasogastric tube was clamped to facilitate imaging. The patient returned to the ward late in the evening when the ward was staffed by agency staff with no experience of management of nasogastric tubes. No instructions were written in the notes to indicate that the tube should have been either left on free drainage or aspirated. During the night, the patient developed severe respiratory distress secondary to aspiration of gastric contents, and despite transfer to the intensive care unit and respiratory intervention, succumbed to aspiration pneumonia.

#### Reporter’s comments

The trust investigated this incident and put the following remedial actions in place:

• In response to concerns about communications of clinical instructions, a structured ward round template was introduced.
• A specific teaching session for ward staff in areas managing nasogastric tubes was prepared for delivery at regular intervals.
• A ‘consultant surgeon of the week’ model was introduced, with a single consultant providing ward cover from Monday to Friday and another covering the weekend.
• The trust induction policy was amended to ensure that temporary agency staff were competent to carry out care for patients allocated to them on a particular shift.

#### CORESS comments

Continuity of care and communication were the key issues here. A checklist protocol for management of nasogastric tubes and a formal handover to ward staff on return from radiology would have been of value. It was noted that similar problems have been reported with chest and spinal drains. A flag placed on the tube with specific instructions would also have been helpful.

## TAVI troubles

### Case 293

A 65-year-old man underwent transcatheter aortic valve implantation (TAVI) in a cardiovascular hybrid theatre. At the end of the procedure, the patient was noted to be hypotensive and tachycardic. Angiography confirmed bleeding from defects in the common and right external iliac artery. Two covered stents were implanted to seal the areas, with apparent cessation of bleeding. The patient remained ventilated and sedated on the cardiac intensive care unit. The procedure was performed by the hospital’s team of interventional cardiologists with support from cardiac surgeons. No information was recorded in the notes concerning vascular examination.

Around 24 hours after the procedure, the vascular surgery team was called because the patient had cyanosis and absent pulses in the right lower limb. The right foot was severely ischaemic with fixed mottling. Femoral exploration confirmed thrombosis of the right common femoral artery. Femoral thromboembolectomy and patch angioplasty was performed, with recovery of the femoral and popliteal pulses. Despite the technical success of the latter intervention, the lower limb remained ischaemic and unsalvageable. The patient required transarticular amputation at the knee level 24 hours later.

#### Reporter’s comments

The clinicians performing the procedure were focused on the technical aspects of the operation and failed to undertake routine examination of the peripheral circulation. Overnight, development of ischaemia was unnoticed so there was a delay in calling the vascular team and a resultant delay in intervention, by which time the lower limb was unsalvageable. Physical examination before and after any surgical intervention remains essential for a good clinical outcome.

#### CORESS comments

A CORESS advisory board member remarked on a recent local audit in which a significant number of patients admitted under medicine or cardiology with an initial cardiac complaint identified that few had documentation of a vascular examination beneath the diaphragm. For patients where intervention has involved instrumentation of lower-limb vessels, the post-interventional surveillance protocols must involve regular assessment of the limb circulation for at least the first 24 hours.

## Straying from a safe plane

### Case 294

An oral and maxillofacial surgical trainer was supervising a novice trainee undertaking an operation that required a mucosal flap to be raised from the lower lip to gain access to the mandible bone. The trainee was inexperienced and quite nervous, and found it difficult to get into the correct plane. Looking from the opposite side, the trainer felt that the trainee was in a safe plane and encouraged them to make the deeper cuts to expose the mandible. Only after taking over and continuing to cut did the trainer realise that the lip had been folded beneath the retractor. After removing the retractor, it was noticed that the skin had been damaged in three places. Fortunately, this damage was relatively minor. The skin wounds were closed and the rest of the operation was completed without issue.

#### Reporter’s comments

The trainer was not able to appreciate that the trainee had strayed from a safe plane because he was not viewing the operation from his normal operating position. In an attempt to reassure and encourage the trainee, the trainer pushed them further and faster than was really necessary. Although a technically simple procedure, there is little margin for error in the lower lip. A ‘pause and reset’ would have allowed the experienced surgeon/trainer to identify that the operation had strayed outside the normal plane and prevented the complication.

#### CORESS comments

A lack of situational awareness was compounded by an unusual vantage point for the trainer. Often, touch sensation is as important as vision in surgical dissection. CORESS agrees with the reporter’s comments about the value of a ‘pause and reset’, which allows time for consideration of potential anatomical risks of dissection.

## Inadvertent removal of ureteric stent

### Case 295

A 46-year-old woman was diagnosed with recurrent ovarian cancer in the vaginal vault 5 months after primary cytoreductive surgery and completion of 6 cycles of adjuvant chemotherapy. Total pelvic exenteration was recommended by the multidisciplinary team. An appropriate date for surgery had to be planned with foresight, taking into consideration her recovery from chemotherapy-induced anaemia, radiological investigations to rule out metastatic disease elsewhere, physical and emotional preparedness for major life-changing surgery, and involvement of a multidisciplinary team (gynaecology, colorectal and urology). In addition, owing to the second wave of COVID-19 in the UK and a mini-outbreak in the surgical ward, surgery was postponed further.

The patient underwent total pelvic clearance with formation of a permanent colostomy and urinary diversion, with an ileal conduit. A delayed start due to a pending preoperative COVID-19 test resulted in the procedure being completed in the evening. By this time, the scrub nurse who had assisted from the start of the surgery had handed over to a colleague once the final count was completed. After the wound was dressed, the scrub nurse inadvertently removed the right ureteric stent from the urostomy. Ureteric stents had been placed prophylactically to avoid a stricture during healing of the anastomosis.

The anaesthetist was immediately requested to continue the administration of general anaesthetic while the consultant urologist was urgently contacted. A flexible cystoscope was passed down the urostomy. The ureteric orifice was visible but cannulation proved to be a challenge. A decision was therefore made to reopen the abdomen. The ureteroileal anastomosis was opened and a guidewire passed to the right kidney before a fresh ureteric stent was inserted into position. The abdomen was then closed for the second time. The patient was debriefed about the entire course of events on the first postoperative day and understood the need to reopen the abdomen. She made an excellent postoperative recovery.

#### Reporter’s comments

Haste to clear up at the end of a long procedure led to unintentional removal of important patient attachments. The late finish of the procedure resulted in change of staff and disruption in continuity of care. A complete handover is imperative to maintain continuity of care irrespective of the length of the procedure. It is preferable to ensure an early start for major surgery so that the same team can continue until the operation is completed. The entire multidisciplinary team should be represented to the end of every procedure. The importance of the placement of ureteric stents should be highlighted and it is preferable for every individual in the team to be mindful of their location. These stents can be up to 30cm in length and can obstruct the surgical field.

#### CORESS comments

This was a complex procedure in which a number of factors conspired to contribute to this adverse incident. A failure to communicate the importance and relative insecurity of the stents to scrub staff was the principal causative factor. It was noted that stents are routinely placed to reduce the effects of oedema/stricture and potential early leakage at the ureteroileal anastomosis.

The CORESS advisory board felt that a member of the urological surgical team should have been present on completion of the procedure to ensure that an appropriate urostomy bag was placed over the ileal conduit spout and stents, preventing their dislodgement. Stents would usually be left in situ for 7–10 days but are not secured with sutures because of the ileal conduit spout. A precautionary comment was made about the potential for inadvertent removal of drains when removing modern adhesive drapes, which may stick to drains. It was also noted in terms of human factors that an intraoperative pause and mini-brief, highlighting key aspects of the procedure, might have helped to focus staff on potential errors. Description of such a system can be found in: Hardie *et al*. Patient, Procedure, People (PPP): recognising and responding to intraoperative critical events. *Ann R Coll Surg Engl* 2022; **104**: 409–413.

## Post-thyroidectomy haematoma

### Case 296

A 58-year-old man was noted to have a large, toxic goitre and was referred to the endocrine team. Thyroidectomy was undertaken with placement of a suction drain. The list started late and the case was finished in the early evening. The patient returned to the ward from recovery at around 10pm. On arrival on the ward, some neck swelling was noted but there was minimal blood in the suction drain bottle and vital signs were normal. In the early hours of the morning, ward staff were alerted when the patient activated his emergency alarm. A nurse attended to find the patient very distressed with a significantly swollen neck. An arrest call was put out and the resuscitation team attended.

The anaesthetist was unable to visualise the vocal cords to insert an endotracheal tube but was able to place a laryngeal mask airway and to oxygenate the patient. The patient was rapidly returned to theatre, where the neck wound was reopened and a large quantity of clot evacuated under general anaesthesia. A tracheostomy was undertaken. The patient was transferred to the intensive care unit overnight. Unfortunately, on being weaned off the ventilator, it became apparent on waking that he had suffered a neurological insult. Computed tomography subsequently confirmed a hypoxic brain injury, from which he did not recover.

#### Reporter’s and CORESS comments

Postoperative haemorrhage after thyroidectomy occurs in 0.45–4.2% of patients, up to a quarter of whom may develop acute airways compromise.^1^ The Difficult Airway Society, the British Association of Endocrine and Thyroid Surgeons, and ENT UK have recently published guidelines on management of haematoma after thyroid surgery.^1^ Eight recommendations have been made, including: training all those who care for post-thyroidectomy patients to look out for signs of bleeding that may compromise the airway; presence of an emergency kit at the bedside of post-thyroidectomy patients, including during transfer; presence of front-of-neck airway equipment, including bougie, scalpel and tracheal tube on wards caring for these patients; development of a systematic approach to reopening the neck at the bedside, where necessary, to relieve the haematoma (SCOOP – skin exposure, cut sutures, open skin, open muscles [superficial and deep layers], pack wound).

The CORESS advisory board noted that a postoperative ward round might have picked up this complication earlier. In extremis, had a surgeon been called to examine the patient on the ward, a decision might have been made to remove clips and decompress the haematoma on the ward rather than incur delay in transferring the patient back to theatre.

Reference1.Iliff
HA, El-Boghdadly
K, Ahmed
I
*et al*. Management of haematoma after thyroid surgery: systematic review and multidisciplinary consensus guidelines from the Difficult Airway Society, the British Association of Endocrine and Thyroid Surgeons and the British Association of Otorhinolaryngology, Head and Neck Surgery. *Anaesthesia*
2022; **77**: 82–95.34545943
10.1111/anae.15585PMC9291554

## Missing drawing pin

### Case 297

During a complex total pelvic exenteration and sacral resection for recurrent rectal cancer, a drawing pin was placed in the patient’s sacrum, within the abdomen, at the cranial limit of the sacral resection (S3), to enable radiological identification of the extent of resection. The patient underwent formation of colonic and ileal conduits, and was then placed prone for the sacrectomy. The sacrum was resected *en bloc* with the tumour, large bowel, bladder and prostate. Flaps were raised and the defect was closed. Later, it was discovered that the pin was not in the resection specimen. The pin had not been included in the instrument and swab count. Subsequent computed tomography localised the pin and a second laparotomy was performed to remove this 48 hours later. The pathology report confirmed that an R0 resection had been achieved and the patient was discharged on postoperative day 6.

#### Reporter’s and CORESS comments

There was lack of communication between the surgeon and scrub staff. The drawing pin should have been included in the count. The scrub team thought that the pin was intended to remain in situ and so the surgeons should have told the scrub team that it was meant to come out with the specimen, especially since during what was a long operation, the scrub team changed twice. Better communication and a full handover might have reduced the risk of this incident occurring. This was a complex case with various factors contributing to the adverse outcome, including failure to add the ‘extra’ kit (the drawing pin) to the count so that it was not counted in or out and the need to turn the patient intraoperatively. Adequate communication and discussion of use of the pin in a pre or intraoperative brief might have prevented this outcome.

## Inadvertent arterial cannulation with PICC line

### Case 298

A 28-year-old woman with recurrent Crohn’s disease was admitted with proximal small-bowel obstruction, vomiting and weight loss, with a body mass index of 18kg/m^2^. It was decided that total parenteral nutrition was needed to improve her nutrition and clinical chemistry before undertaking surgical resection of the affected bowel. A peripherally inserted central catheter (PICC) line was placed for this purpose, using the right brachial vein, and chest radiography was performed to check the position of the line.

The following morning, the patient complained of a cold right hand and paraesthesia. No radial pulse could be palpated. A vascular opinion was asked for immediately but there was a request for Doppler ultrasonography to be undertaken first. The Doppler imaging took two hours to be performed. The Doppler indicated that the catheter was lying in the brachial artery and had then been fed into the arch of the aorta. The patient was taken to theatre, the catheter removed and brachial thrombectomy undertaken with vein patch closure. The arterial supply was re-established and a further feeding line was placed approximately 4–5 hours after the injury was discovered. The complex Crohn’s disease was operated on three weeks later with a successful outcome. The patient’s right hand remained warm with normal pulses and normal sensation.

#### Reporter’s comments

The PICC line was inadvertently placed into the brachial artery, and the difference between arterial and venous blood was not recognised at the time of placement. The nursing team did not recognise the issue and escalate this to the medical team. The vascular team did not see the patient until after the Doppler ultrasonography although the clinical signs had suggested an arterial injury and ischaemia. This delayed correction of the problem of acute ischaemia by four hours and might have exacerbated any reperfusion injury, potentially even requiring forearm fasciotomies.

#### CORESS comments

In potential acute limb ischaemia, the patient should undergo rapid clinical assessment by an appropriate clinician and then, if necessary, appropriate investigations can be obtained (not vice versa). In this case, use of ultrasonography to facilitate line placement would probably have averted incorrect line siting.
